# Efficacy of modified LiuJunZi decoction on functional dyspepsia of spleen-deficiency and qi-stagnation syndrome: a randomized controlled trial

**DOI:** 10.1186/1472-6882-13-54

**Published:** 2013-03-02

**Authors:** Shengsheng Zhang, Luqing Zhao, Hongbing Wang, Chuijie Wang, Suiping Huang, Hong Shen, Wei Wei, Lin Tao, Tao Zhou

**Affiliations:** 1 Beijing Hospital of Traditional Chinese Medicine Affiliated to Capital Medical University, No. 23 Meishuguan Back Street, Dongcheng District, Beijing, 100010, China; 2 The Affiliated Hospital of Liaoning University of Traditional Chinese Medicine, No. 33 Beiling Street, Huanggu District, Shenyang, 110033, China; 3 The Second Affiliated Hospital of Guangdong University of Traditional Chinese Medicine, No. 111 Dade Street, Baiyun District, Guangzhou, 510120, China; 4 The Affiliated Hospital of Nanjing University of Traditional Chinese Medicine, No. 155 Hanzhong Street, Jianye District, Nanjing, 210029, China; 5 Wangjing Hospital, Huajiadi Street, Chaoyang District, Beijing, 100102, China

**Keywords:** Functional dyspepsia, Chinese herbal medicine, Modified LiuJunZi decoction, Randomized controlled trial

## Abstract

**Background:**

Chinese herbal medicine (CHM) has been used in China and some other countries for the treatment of patients with functional dyspepsia (FD). However, controlled studies supporting the efficacy of such treatments in patients with FD are lacking. In this trial, we aimed to assess the efficacy and safety of modified LiuJunZi decoction in patients with FD of spleen-deficiency and qi-stagnation syndrome.

**Methods:**

We performed a randomized, double-blind, placebo-controlled trial with patients from five centers. Patients with FD of spleen-deficiency and qi-stagnation syndrome (n = 160) were randomly assigned to groups given CHM modified LiuJunZi decoction or placebo in a 2:1 ratio. Herbal or placebo granules were dissolved in 300 ml of boiled water cooled to 70°C. Patients in both groups were administered 150 ml (50°C) twice daily. The trial included a 4-week treatment period and a 4-week follow-up period. The primary outcomes were dyspepsia symptom scores, measured by the total dyspepsia symptom scale and the single dyspepsia symptom scale at weeks 0, 1, 2, 3, 4 and 8. The secondary outcome was the change of radiopaque barium markers emptied from the stomach between week 0 and week 4 of treatment.

**Results:**

Compared with patients in the placebo group, patients in the CHM group showed significant improvements according to the scores of total dyspepsia symptoms and single dyspepsia symptoms obtained from patients (P < 0.01) and investigators (P < 0.01). They also showed an improvement in the number of radiopaque barium markers emptied from the stomach (P < 0.05).

**Conclusions:**

CHM modified LiuJunZi decoction appears to offer symptomatic improvement in patients with FD of spleen-deficiency and qi-stagnation syndrome.

**Trial registration:**

Chinese Clinical Trial Registry (ChiCTR): http://ChiCTR-TRC-10001074

## Background

Functional dyspepsia (FD) is a common functional gastrointestinal disorder characterized by chronic or recurrent upper abdominal fullness, epigastric pain, belching, bloating, early satiety, nausea, vomiting, regurgitation, burning, loss of appetite, and other symptoms. FD accounts for a significant proportion of patients seen in gastroenterology offices. The global prevalence of FD is estimated between 11.5% and 29.2% [1-4]. The direct and indirect economic burden caused by FD is huge and has considerable negative impact on productivity [5,6]. The pathophysiology of FD is poorly understood, although various mechanisms are thought to play a role in the development of symptoms [7-10]. No single available treatment is reliably effective for this condition. Many studies have suggested the potential effectiveness of Chinese herbal medicine (CHM) in the treatment of FD [11], but most of the previous clinical trials have lacked rigor and used poor techniques for randomization and blinding. To date, no strong scientific evidence supporting the use of CHM in FD is available.

In Traditional Chinese Medicine (TCM), FD is considered nearly equivalent to the TCM term “stuffiness and fullness” [12], which is divided into different syndromes according to the clinical symptoms and signs. In our previous research, we studied the distribution of the different syndromes in 565 cases of FD and found that “spleen-deficiency and qi-stagnation” is the most common syndrome in FD patients [13]. The Chinese consensus on diagnosis and treatment of functional dyspepsia also defines spleen-deficiency and qi-stagnation as a prominent syndrome in FD [14]. LiuJunZi decoction is a traditional Chinese compound herbal recipe for invigorating the spleen and regulating qi. We added the related herbal medicines Cortex Mangnoliae officinalis, Common Vladimiria Root, Rhizoma Corydalis, and Villous amomrum fruit to the recipe, and found a modified LiuJunZi decoction with a satisfactory clinical effect. Previous studies had shown that the active ingredients in the modified LiuJunZi decoction can improve gastrointestinal motility, regulate gastrointestinal function and have anti-Helicobacter pylori and anti-inflammatory action [15-21].

In this trial, we aimed to test the efficacy of the modified LiuJunzi decoction in patients with FD and spleen-deficiency and qi-stagnation syndrome using a randomized, double-blind, placebo-controlled study design.

## Methods

### Design

This study was a double-blind, placebo-controlled clinical trial. Patients were randomized into CHM or placebo groups in a 2:1 ratio. Because it would be unethical to assign an equal number of ill subjects to the ineffective placebo treatment, the 2:1 randomization plan was chosen to protect the rights of the subjects. The trial protocol was approved by regional ethics review boards, including the National Review Board for Clinical Drug Research in the Beijing Hospital of Chinese Medicine Hospital affiliated to Capital Medical University and the Affiliated Hospital of Nanjing University of Traditional Chinese Medicine. There were no major changes in the study protocol after initiation of the study.

### Participants

Patients were screened by investigators at five sites in China: the Beijing Hospital of Traditional Chinese Medicine affiliated to Capital Medical University, the Affiliated Hospital of Liaoning University of Traditional Chinese Medicine, the Second Affiliated Hospital of Guangdong University of Traditional Chinese Medicine, the Affiliated Hospital of Nanjing University of Traditional Chinese Medicine, and the Beijing Xuanwu Hospital of Traditional Chinese Medicine. The study was conducted between April 2009 and February 2011. Patients were assessed according to the Rome III criteria and the Guiding principle for clinical research on new drugs of traditional Chinese medicine [12]. The inclusion and exclusion criteria are shown in Table 1. Written informed consent was obtained from all patients prior to inclusion in the trial. Patients were free to withdraw from the study at any time.

**Table 1:** Inclusion, exclusion and spleen-deficiency and qi-stagnation syndrome diagnostic criteria

**Inclusion criteria**	1. Patients who meet the Rome III diagnosis standard of functional dyspepsia.
	2. Patients who have Spleen-deficiency and qi-stagnation syndrome.
	3. Patients aged 18 to 65 without gender limitation.
	4. Signed the informed consent.
**Exclusion criteria**	1. Patients who combined with GI ulcer, erosive gastritis, atrophic gastritis, severe dysplasia of gastric mucosa or suspicious malignant lesion.
	2. Patients who have overlap syndrome combined with gastroesophageal reflux disease or irritable bowel syndrome.
	3. Patients whose syndrome is difficult to differentiate.
	4. Patients who have connective tissue diseases, diabetes or other endocrine disease, climacteric syndrome, or severe diseases in heart, liver, lung, kidney, blood.
	5. Pregnant or lactating women. Disabled people.
	6. Patients with history of alcoholic or drug abuse.
	7. Patients who have allergic constitution or known to be allergic to the drug used in this trial.
	8. Patients who are involved in other trials.
	9. Patients with poor compliance or other reasons that the researcher considered not to be appropriate to participate in this trial.
	10. Patients with severe depression and have suicidal tendency.
**Spleen-deficiency and qi-stagnation syndrome diagnostic criteria**	The spleen-deficiency and qi-stagnation syndrome is defined as having the main symptoms and at least two of the accompanying symptoms, as well as pale tongue with whitish tongue coating and deep and thready pulse. The main symptoms include epigastric stuffiness and fullness, and asthenia. While the accompanying symptoms include epigastric stuffiness and fullness aggravated after meal, epigastric pain, decreased appetite, belching and acid regurgitation, fullness and discomfort in chest and hypochondrium, nausea and vomiting, and constipation or loose stool.

### Randomization and blinding

Randomization was performed with SAS9.10. Eligible patients were assigned a randomization number according to a predetermined list at each center. These numbers were allocated to patients in sequential order and registered in the patient enrolment list and the allocation was concealed. Emergency envelopes containing the randomization code were provided to the investigators and were examined at the end of the trial to ensure that the blinded conditions had been maintained.

### Interventions

Patients in CHM group were provided granules of Chinese herbal extracts, which were prepared by Tcmages Pharmaceutical Co., Ltd. (Beijing, China). The standard herb formula (Table 2) was a modified LiuJunZi decoction. Patients in the placebo group were given placebo granules, which were prepared by the same supplier and were designed to taste, smell and look similar to the Chinese herbal formula granules. Granules were dissolved in 300 ml boiled water cooled to 70°C. Patients in both groups were required to take 150 ml (50°C) twice daily. For the duration of the trial, the patients were not allowed to take any concomitant medications associated with the treatment of FD. Treatment continued for 4 weeks and was followed by a 4-week follow-up period.

**Table 2:** Chinese herb formula

**Chinese name**	**Pharmaceutical name**	**Powdered herb, %**
Dang Shen	Pilose Asiabell Root	16.5
Bai Zhu	Largehead Atractylodes Rhizoma	11.0
Fu Ling	Indian Buead	11.0
Gan Cao	Liquorice Root	5.5
Hou Po	Cortex Mangnoliae officinalis	11.0
Mu Xiang	Common Vladimiria Root	11.0
Sha Ren	Villous Amomrum Fruit	6.5
Yuan Hu	Rhizoma Corydalis	16.5
Ban Xia	Pinellia Tuber	11.0

### Outcomes

#### Primary outcome

We assessed FD symptoms using two scales: 1) the total dyspepsia symptoms scale and 2) the single dyspepsia symptom scale. Ratings were completed by both the investigators and patients at baseline and at weeks 1, 2, 3, 4 and 8.

##### Total dyspepsia symptom scale (TDS)

The TDS scale consisted of the assessment of eight items (postprandial fullness and bloating, early satiety, epigastric pain, epigastric burning, nausea, vomiting, belching and “other symptoms”), each with four options (absent = 0, mild = 1, moderate = 2, or severe = 3).

##### Single dyspepsia symptom scale (SDS)

The SDS scale measured three aspects of four principal symptoms of FD. The symptoms were epigastric pain, epigastric burning, postprandial fullness and bloating, and early satiety. The three aspects were the frequency, intensity and level of discomfort, and were rated by four options (absent = 0, mild = 1, moderate = 2, or severe = 3). The total score obtained using this scale was called the single dyspepsia symptom (SDS) score.

#### The secondary outcome

##### Gastric emptying trial using radiopaque barium markers

Patients ate the standard meal and 20 barium markers after fasting for 6–8 hours. The standard meal consisted of a bag of instant noodles (97 g) and pork intestines (45 g). Abdominal radiographs were taken at 5 hours after eating, and the number of residual markers in the stomach was counted. The number of radiopaque barium markers emptied from the stomach was used as a measure of gastric emptying.

### Safety monitoring

To assess the safety of the 4-week treatment, routine tests of blood, urine and stool samples, as well as electrocardiogram (ECG) and blood biochemical tests (ALT, AST, BUN, Scr), were conducted before randomization and immediately after the completed treatment. During the trial, adverse events were observed in detail and documented using case report forms (CRFs).

### Sample size

We performed sample size calculations in two ways. To guarantee the reliability of the trial, the calculation yielding the larger sample size was used. The sample size was calculated according to the following formula [22]:

$$\begin{array}{l} n_{1}=\frac{{\left[u_{\alpha }\sqrt{\pi_{c}\left(1-\pi_{c}\right)\left(1+c\right)/c}+u_{\beta }\sqrt{\pi_{1}\left(1-\pi_{1}\right)+\pi_{2}\left(1-\pi_{2}\right)/c}\right]}^{2}}{{\left(\pi_{1}-\pi_{2}\right)}^{2}}\\ n_{2}=cn_{1}\\ n_{1}{\mathrm{CHM},n}_{2}\mathrm{placebo}\\ \pi_{c}=\frac{\pi_{1}+c\pi_{2}}{1+c},\;u_{\alpha }=1.64,u_{\beta }=1.28,\\ c=2,\pi_{1}=0.5,\pi_{2}=0.75 \end{array}$$

The patients were assigned to either the CHM group or the placebo group (in a 2:1 ratio). The effective rates of treatment and placebo were assumed to be 75% and 50%, respectively [23,24]. The calculation indicated that a sample size of 138 would be sufficient (n = 92 in the treatment group, n = 46 in control group). To allow for a 15% rate of dropouts and missing data, we recruited 106 patients for the treatment group and 54 patients for the control group.

### Statistical analysis

We used intention to treat (ITT) analyses, using all available data at each time point and the baseline observation carried forward (BOCF) approach for missing data. The statistical analysis was performed by the Center of Clinical Epidemiology of the Third Hospital of Peking University. Parametric Student’s t-tests or non-parametric Wilcoxon tests were used to quantitatively compare variables, according to distribution characteristics. Quantitative variables are reported as mean ± SD. Statistical significance was considered at P < 0.05.

## Results

### Study population

Between April 2009 and February 2011, a total of 160 patients were recruited: 106 were randomized into the CHM group and 54 into the placebo group. Seven patients withdrew from the trial due to a lack of efficacy. No adverse events were reported. The physiological tests obtained after 4 weeks of treatment showed no abnormal values.

### Participant flow

**Figure 1:**
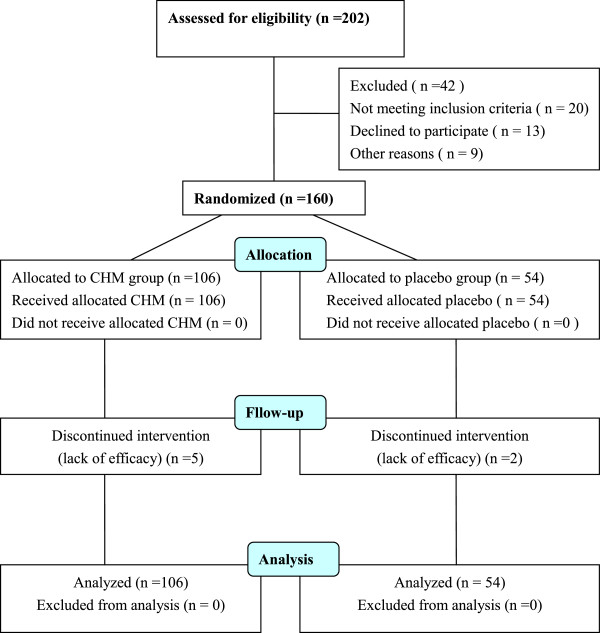
The flow of participants in the study.

The flow of participants in the study is summarized in Figure 1.

### Baseline data

The general characteristics of the patients are shown in Table 3. No significant differences were identified between the two groups in parameters such as gender, age, course of disease or symptom scores before treatment.

**Table 3:** Baseline date of two groups

**Variables**	**CHM (n = 106)**	**placebo (n = 54)**	**P Values**
Characteristic			P > 0.05
Age(year)	39.48 ±13.75	41.63 ± 12.31	
Sex ratio(male:female)	35:71	15:39	
Height(Cm)	164.94 ± 7.41	164.57 ± 7.01	
Weight(Kg)	58.25 ± 10.04	58.91 ± 9.85	
Course of disease(month)	43.24 ± 51.41	45.30 ± 65.42	
TDS and SDS scores (week 0)			
Gastroenterologist TDS scores	6.58 ± 2.23	6.61 ± 2.11	
Patient TDS scores	6.56 ± 2.18	6.81 ± 2.31	
Gastroenterologist SDS scores			
Epigastric pain	3.32 ± 2.27	2.85 ± 2.10	
Epigastric burning	0.98 ± 1.74	0.96 ± 1.67	
Postprandial fullness and bloating	5.04 ± 1.97	5.25 ± 1.96	
Early satiety	3.35 ± 2.39	3.69 ± 2.33	
Patient SDS scores			
Epigastric pain	3.25 ± 2.28	2.96 ± 2.27	
Epigastric burning	1.00 ± 1.76	1.02 ± 1.79	
Postprandial fullness and bloating	5.02 ± 1.93	5.19 ± 1.94	
Early satiety	3.33 ± 2.37	3.76 ± 2.44	

### Primary outcome variables

#### Total dyspepsia symptoms scale

**Figure 2:**
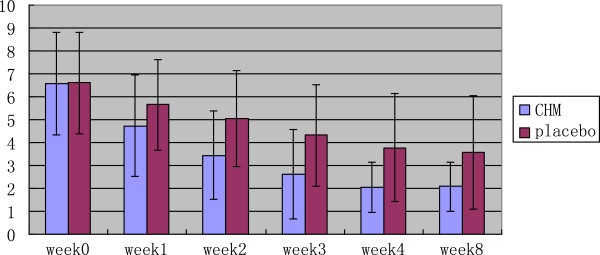
TDS score-change (by gastroenterologists).

**Figure 3:**
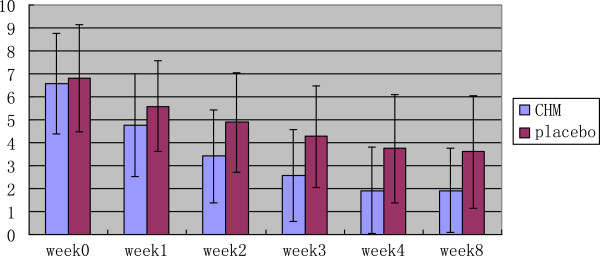
TDS score-change (by patients).

After 4 weeks of treatment, the TDS score assessed by investigators was significantly better for the CHM group than for placebo (Z = −4.629, P < 0.01). At week 8, the score was also significantly better for CHM than for placebo (Z = −3.676, P < 0.01). The TDS scores provided by the patients themselves were similar to those given by the investigators (Table 4, Figures 2 and 3).

**Table 4:** TDS and SDS scores

**Variables**	**CHM (n = 106)**	**placebo (n = 54)**	**P Values**
Week 1			
Gastroenterologist TDS scores	4.73 ± 2.21	5.65 ± 1.96	0.010
Patient TDS scores	4.75 ± 2.24	5.59 ± 1.97	0.021
Gastroenterologist SDS scores			
Epigastric pain	2.47 ± 1.91	2.61 ± 1.99	0.668
Epigastric burning	0.74 ± 1.42	1.09 ± 1.86	0.218
Postprandial fullness and bloating	3.70 ± 1.99	4.68 ± 2.02	0.004
Early satiety	2.42 ± 2.16	3.39 ± 2.12	0.008
Patient SDS scores			
Epigastric pain	2.42 ± 1.93	2.61 ± 1.99	0.568
Epigastric burning	0.73 ± 1.42	1.11 ± 1.88	0.189
Postprandial fullness and bloating	3.73 ± 2.03	4.63 ± 2.05	0.009
Early satiety	2.39 ± 2.16	3.33 ± 2.06	0.009
Week 2			
Gastroenterologist TDS scores	3.44 ± 1.93	5.04 ± 2.11	0.000
Patient TDS scores	3.42 ± 2.02	4.89 ± 2.17	0.000
Gastroenterologist SDS scores			
Epigastric pain	1.74 ± 1.81	2.02 ± 1.80	0.350
Epigastric burning	0.58 ± 1.33	0.85 ± 1.50	0.271
Postprandial fullness and bloating	2.92 ± 1.66	4.24 ± 1.89	0.000
Early satiety	1.82 ± 1.93	2.85 ± 2.03	0.003
Patient SDS scores			
Epigastric pain	1.69 ± 1.79	1.96 ± 1.78	0.360
Epigastric burning	0.59 ± 1.35	0.85 ± 1.50	0.291
Postprandial fullness and bloating	2.92 ± 1.66	4.26 ± 1.85	0.000
Early satiety	1.83 ± 1.94	2.85 ± 2.02	0.003
Week 3			
Gastroenterologist TDS scores	2.63 ± 1.95	4.31 ± 2.23	0.000
Patient TDS scores	2.57 ± 1.98	4.28 ± 2.22	0.000
Gastroenterologist SDS scores			
Epigastric pain	1.11 ± 1.65	1.74 ± 1.70	0.028
Epigastric burning	0.27 ± 0.83	0.70 ± 1.34	0.034
Postprandial fullness and bloating	2.15 ± 1.88	3.87 ± 1.96	0.000
Early satiety	1.25 ± 1.82	2.59 ± 1.99	0.000
Patient SDS scores			
Epigastric pain	1.11 ± 1.65	1.70 ± 1.71	0.039
Epigastric burning	0.30 ± 0.87	0.70 ± 1.34	0.049
Postprandial fullness and bloating	2.13 ± 1.89	3.83 ± 1.96	0.000
Early satiety	1.19 ± 1.81	2.57 ± 1.95	0.000
Week 4			
Gastroenterologist TDS scores	2.03 ± 1.09	3.78 ± 2.35	0.000
Patient TDS scores	1.92 ± 1.89	3.74 ± 2.35	0.000
Gastroenterologist SDS scores			
Epigastric pain	0.92 ± 1.54	1.41 ± 1.65	0.061
Epigastric burning	0.20 ± 0.75	0.56 ± 1.22	0.020
Postprandial fullness and bloating	1.60 ± 1.87	3.35 ± 2.04	0.000
Early satiety	0.83 ± 1.55	2.17 ± 1.98	0.000
Patient SDS scores			
Epigastric pain	0.90 ± 1.53	1.41 ± 1.65	0.040
Epigastric burning	0.20 ± 0.75	0.56 ± 1.22	0.020
Postprandial fullness and bloating	1.61 ± 1.87	3.35 ± 2.03	0.000
Early satiety	0.79 ± 1.54	2.15 ± 1.99	0.000
Week 8			
Gastroenterologist TDS scores	2.08 ± 1.07	3.57 ± 2.46	0.000
Patient TDS scores	1.92 ± 1.84	3.61 ± 2.46	0.000
Gastroenterologist SDS scores			
Epigastric pain	0.87 ± 1.59	1.52 ± 1.60	0.005
Epigastric burning	0.20 ± 0.75	0.48 ± 1.18	0.084
Postprandial fullness and bloating	1.64 ± 1.78	3.28 ± 2.24	0.000
Early satiety	0.70 ± 1.47	1.83 ± 2.08	0.000
Patient SDS scores			
Epigastric pain	0.86 ± 1.58	1.52 ± 1.60	0.004
Epigastric burning	0.20 ± 0.75	0.48 ± 1.18	0.084
Postprandial fullness and bloating	1.61 ± 1.80	3.20 ± 2.24	0.000
Early satiety	0.73 ± 1.51	1.94 ± 2.04	0.000

The results were clinically meaningful. Ratings of the clinical global impression of improvement after the treatment showed the following significant results for the treatment group vs. placebo group: very much improved (50.9% vs. 18.5%), much improved (22.6% vs. 29.6%), small improvement (18.9% vs. 14.8%), and unchanged or deterioration (7.5% vs. 37.0%) (*χ*2 = 26.559; P < 0.001).

#### Single dyspepsia symptom scale

SDS scores assessed by investigators: After 4 weeks of treatment, the scores of postprandial fullness and bloating, early satiety, and burning sensation were significantly better for the CHM group than for placebo (P < 0.01 or P < 0.05). The score of epigastric pain was not different between the two groups (P > 0.05). At week 8, the scores of epigastric pain, postprandial fullness, and early satiety were significantly better for CHM than for placebo (P < 0.01). The score of burning sensation was not different between the two groups (P > 0.05) (Table 4).

The SDS scores provided by patients were similar to those given by investigators. At week 4, the gastroenterologists’ scores of postprandial fullness and bloating were lower than the patients’ scores, whereas the gastroenterologists gave higher scores for epigastric pain and early satiety than the patients did. At week 8, the scores for early satiety given by the gastroenterologists were higher than those given by patients.

### The secondary outcome

#### Gastric emptying using radiopaque barium markers

After CHM treatment, the number of radiopaque barium markers emptied from the stomach from week 0 to 4 was significantly higher in the CHM group (5.16 ± 7.53) compared with the placebo group (1.50 ± 3.21) (P = 0.036).

## Discussion

Functional dyspepsia is a heterogeneous disorder. It involves many pathogenic factors and different pathophysiological disturbances, including delayed gastric emptying, impaired accommodation, and hypersensitivity to gastric distention. Treatment of the underlying pathophysiological abnormality seems logical, but the main pharmacotherapeutic options include acid suppression, prokinetic drugs, and antidepressants [6,25-27], all of which have limited effects. Herbal formulations are widely used to treat FD in China and many other areas in the world. However, the available evidence of the efficacy of these formulas is inadequate.

This multi-center, randomized, double-blind, placebo-controlled study indicates that modified LiuJunZi decoction is effective in the management of symptoms associated with FD and gastric emptying. The effects appeared to last for up to 4 weeks after completion of treatment, and were particularly beneficial for postprandial fullness and bloating and early satiety. Patients receiving modified LiuJunZi decoction treatment demonstrated significantly better outcomes (both clinically and statistically) on all the outcome measures compared with patients receiving placebo. Moreover, no adverse events were reported during the study.

The evaluation of treatment effects in FD is difficult and there is currently no gold standard. In our study, we used two different parameters as the main target variables. The TDS scale included almost all symptoms associated with FD, and the SDS scale included information on the four principal symptoms of FD, measured in terms of the frequency, intensity and level of discomfort. The main target variables were recorded by both investigators and patients. We also assessed gastric emptying with radiopaque barium markers as a more objective outcome. Another difficulty in clinical trials with patients with FD is the remarkable placebo response. It has been shown that one third of patients with FD will respond to placebo in short-term trials [28], and the proportion may be even higher in long-term studies. In our study, we made a great effort to make the treatments in the two groups indistinguishable for the patients. A placebo of similar appearance, smell and taste to the active concoction was used. To ensure that the patients were not able to discriminate between placebo and active treatment, 20 healthy volunteers participated in a randomized taste and visual assessment of the placebo and active medication. Eight volunteers correctly identified the active compound as active, whereas twelve volunteers considered the placebo preparation to be the active compound. Thus, it is reasonable to assume that the medication was given in an appropriately blinded manner. Despite the well-known high response rate to placebo in FD, we found significantly greater improvements in dyspepsia symptoms and gastric emptying in patients receiving the CHM compared with placebo-treated patients.

In TCM, injury by food or drink, emotional injury, and congenital defects are main pathogenic factors of FD. All these pathogenic factors cause abnormal function of the upper abdomen, spleen and stomach. Spleen-deficiency and qi-stagnation exist throughout the course of the disease. The herbal formula provided to patients in this study was a modified LiuJunZi decoction. All the herbs matched well, and could replenish the deficiencies, and harmonize and regulate the qi. Because the function of the spleen and stomach recovered, all the dyspepsia symptoms were abated and the gastric emptying improved. This is accordance with previous studies [15-21] which showed physiological effects of LiuJunZi decoction and some other herbal medicines in the modified LiuJunZi decoction. In Japan, LiuJunZi decoction is used for functional dyspepsia and has been found to improve the symptoms in patients, regulation of gastrointestinal function and hormone secretion [29,30]. However, herbal preparations are complex and contain a number of active ingredients that may work together. The multiple effects of different active ingredients may be of benefit for the variety of different symptoms that occur in functional gastrointestinal disorders. However, more studies are needed to explore the mechanisms of action and properties of the identified components. FD is a common, chronic and recurrent functional gastrointestinal disorder. This study used a short treatment period and follow-up and a relatively small number of patients, so there is ample room to enhance the evaluation of efficacy and safety by further studies.

## Conclusion

We conclude that modified LiuJunZi decoction may offer symptomatic improvements in patients with FD. In this randomized, double-blinded, placebo-controlled trial, modified LiuJunZi decoction was shown to be effective in the management of FD. Further studies are needed to determine the precise mechanisms of action.

## Competing interests

The authors declare that they have no competing interests.

## Authors’ contributions

ZSS, WHB, ZLQ, LT and ZT contributed to the conception and design of the study. ZSS and ZLQ drafted the manuscript. All authors contributed to further writing of the manuscript. All authors read and approved of the final manuscript.

## Pre-publication history

The pre-publication history for this paper can be accessed here:

http://www.biomedcentral.com/1472-6882/13/54/prepub
